# The Role of Portal Vein Pulsatility and Point-of-Care Ultrasound (POCUS) to Guide Decongestion and Hemodynamic Management in Acute Leptospirosis

**DOI:** 10.7759/cureus.87055

**Published:** 2025-06-30

**Authors:** Keevan Singh, Kevin Singh, Laken Boochoon

**Affiliations:** 1 Department of Anaesthesia and Intensive Care, San-Fernando General Hospital, San Fernando, TTO; 2 Department of Internal Medicine, San-Fernando General Hospital, San Fernando, TTO

**Keywords:** fluid balance, hemodynamic stability, leptospirosis, point-of-care ultrasound (pocus), portal vein pulsatility, venous excess ultrasound score (vexus)

## Abstract

Leptospirosis is a relatively common tropical disease that can present with multiorgan failure. Given its wide range of phenotypes, leptospirosis patients presenting with cardiac and renal failure may benefit from diuresis and decongestion. Estimating left atrial pressure and its surrogates, point-of-care ultrasound (POCUS) can help guide decongestive and hemodynamic therapy in these patients. A novel component of POCUS assessment, portal vein pulsatility, can provide information on end-organ congestion and aid in the titration of hemodynamic interventions.

## Introduction

Leptospirosis is a tropical zoonosis that can lead to pulmonary, renal, and cardiac dysfunction [1]. In its severe form, ICU admission is often needed, with a mortality rate of up to 50% [1]. Tropical diseases such as leptospirosis can comprise up to 30% of ICU admissions in tropical countries [2]. Due to its protean manifestations, supportive therapy is often challenging, and treatment generalizations become difficult [1,2]. Point-of-care ultrasound (POCUS) can allow for personalized care, prevent harm from excessive fluid administration, and guide the use of vasoactive medications [3]. However, there is little published data on the role of POCUS in leptospirosis, likely due to its high acquisition cost and training requirements. In their general review of the use of POCUS in infectious disease, Diez-Vidal, while advocating for its use, limited the use of POCUS to qualitative parameters [3].

POCUS can provide objective determination of cardiac output and identify markers of venous congestion and fluid tolerance [4,5]. Determining fluid tolerance can help guide hemodynamic therapy and interventions to prevent and treat fluid overload, which is associated with increased hospital mortality and organ dysfunction [5,6].

Portal vein pulsatility is a novel quantitative POCUS parameter that can prove useful in guiding hemodynamic management in patients with shock [7]. A high portal vein pulsatility index can indicate significant venous congestion and poor tolerance to intravenous fluid therapy [8]. The combination of multiple POCUS parameters can provide a more comprehensive picture in patients presenting with shock and multiorgan failure. We present an illustrative case where the use of quantitative and qualitative POCUS parameters guided hemodynamic management in a patient presenting with severe leptospirosis and multiorgan failure.

## Case presentation

A 37-year-old previously well female presented to the ED with a short history of vomiting, diarrhea, shortness of breath, and fever. On examination, crepitations were heard in both lung fields with bilateral pitting edema up to her mid-tibia. Blood investigations revealed a serum creatinine level of 36 mg/dL, a blood urea nitrogen level of 140 mg/dL, a potassium level of 8.1 mmol/L, and significant transaminitis. POCUS in the ED revealed poor cardiac contractility with an estimated ejection fraction of 30%. Cardiac troponin T was elevated (0.17 ng/mL), and her ECG showed a sinus tachycardia with a prolonged QT interval and T wave flattening over the anterolateral leads. A CT scan of her kidney revealed no obstructive pathology. Leptospirosis IgM was requested and reported as positive on day 2 of admission.

Despite initial treatment of her hyperkalemia, her metabolic status worsened rapidly (venous blood gas: pH, 6.76; base excess, -30; lactate, 7.4), and she eventually progressed to a cardiac arrest 12 hours after admission. The return of spontaneous circulation was achieved after five cycles of CPR. She was intubated and placed on mechanical ventilation, started on a noradrenaline infusion (0.1 μg/kg/min), and transferred to the ICU.

In the ICU, the noradrenaline infusion was continued, targeting a mean arterial pressure (MAP) of greater than 65 mmHg. A radial arterial line and a left internal jugular central venous catheter were inserted, with a right internal jugular hemodialysis catheter placed for urgent continuous renal replacement therapy (CRRT). She was placed on mechanical ventilation (volume-synchronized intermittent mandatory ventilation with the following settings: tidal volume, 450 mL; breath rate, 12 breaths/min; positive-end expiratory pressure, 8 cmH2O; pressure support, 5 cmH2O) and sedated with a propofol infusion, with the rate adjusted between 50 and 120 mg/hr. Furosemide 60 mg IV was given, and a 100 mL infusion of 8.4% sodium bicarbonate was started. Ceftriaxone 1 g was administered intravenously stat and then once daily.

**Figure 1:**
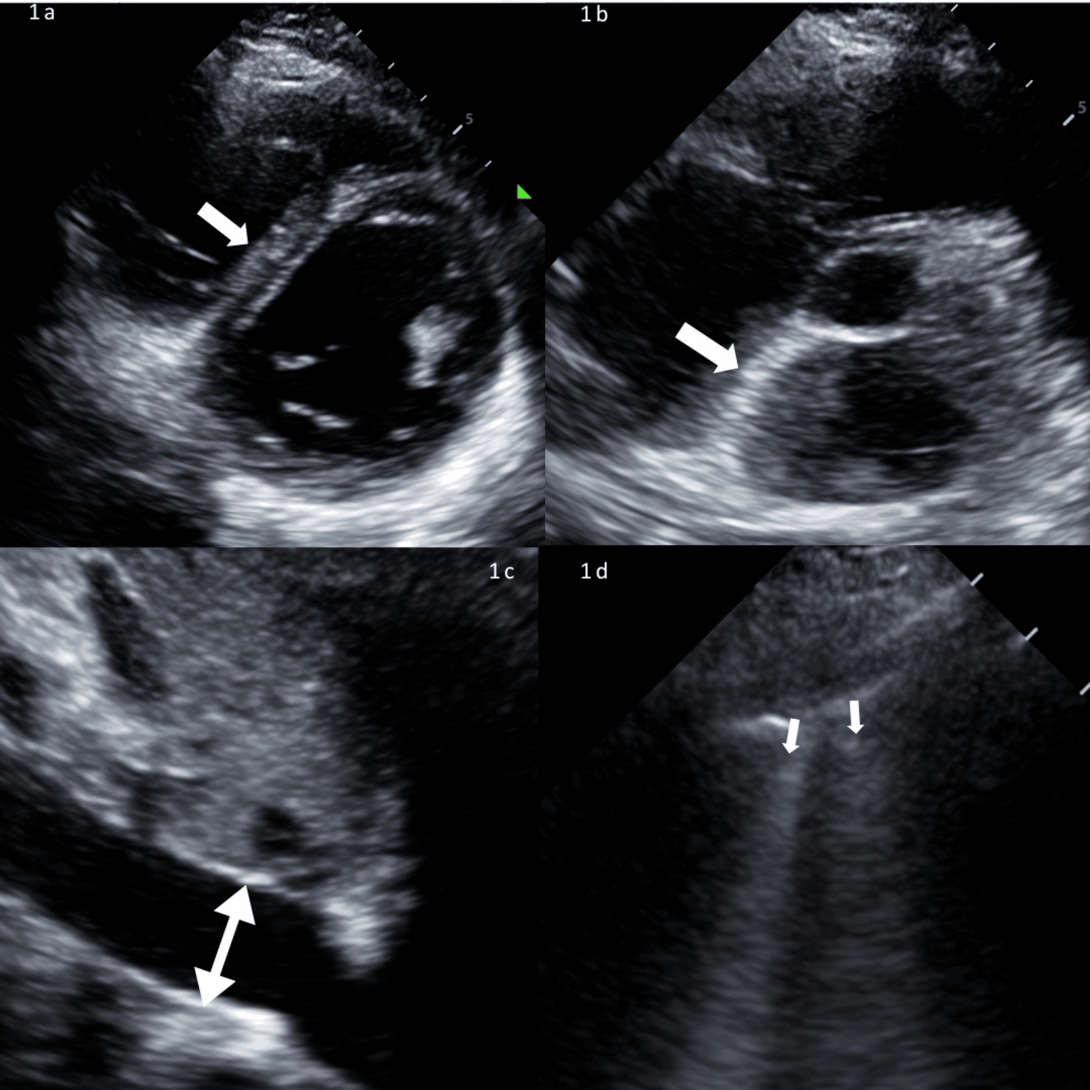
Signs of raised left atrial pressure and organ congestion 1a: Parasternal short-axis view showing flattened D-shaped interventricular septum (white arrow) with paradoxical movement in diastole. 1b: Parasternal short-axis view showing deviated interatrial septum to the right (white arrow). 1c: Inferior vena cava, subcostal view showing a dilated inferior vena cava (2.4 cm) with minimal respiratory variation (white arrow). 1d: Lung ultrasound, right upper quadrant showing B lines (white arrow).

**Figure 2:**
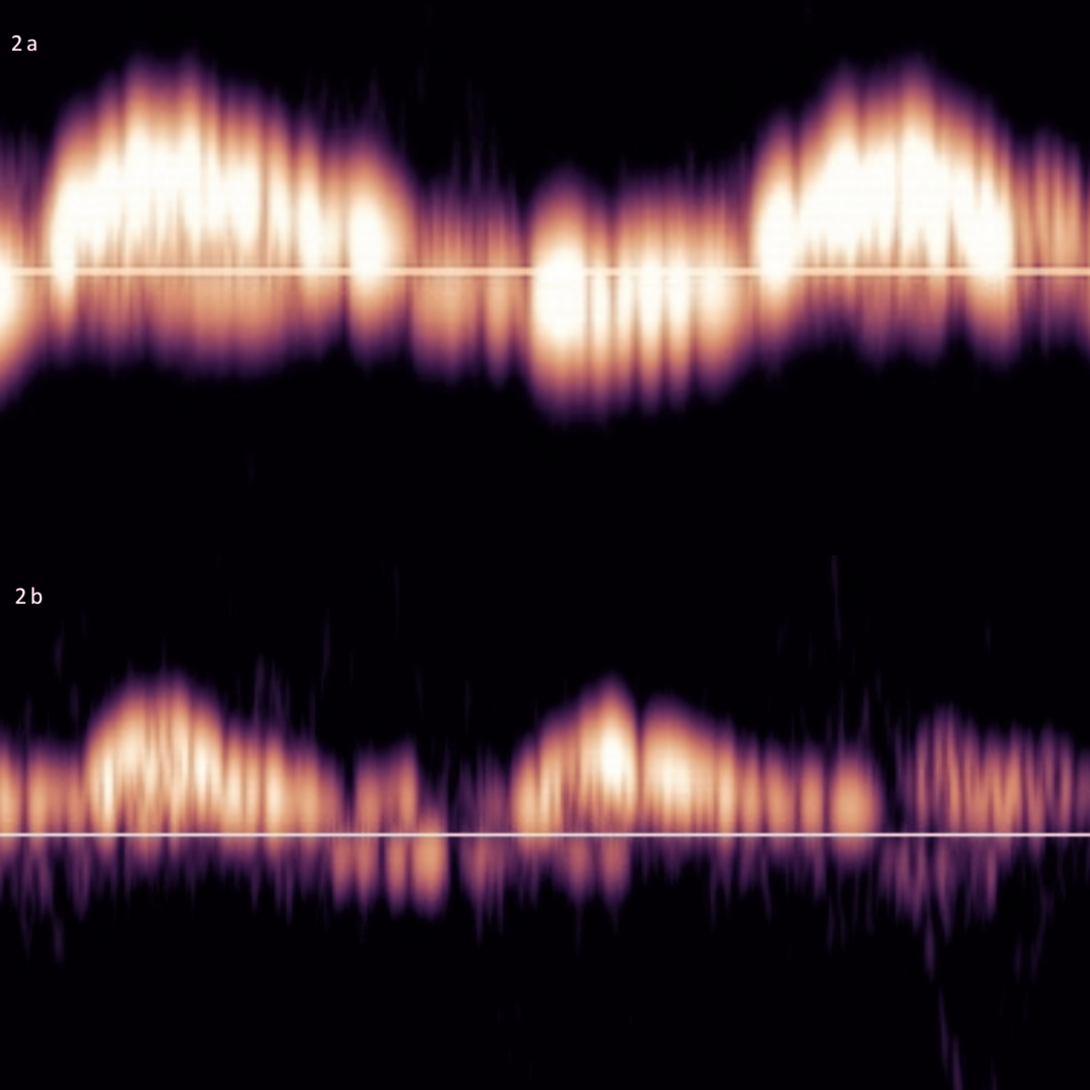
Portal vein Doppler 2a: Initial portal vein Doppler showing a >100% pulsatile Doppler waveform with flow below the baseline noted. 2b: Repeat portal vein Doppler waveform following initial fluid loss of 2400 mL with a pulsatility index of approximately 100% and significantly reduced flow below the baseline being noted.

In the ICU, she had persistent hypoperfusion with a prolonged capillary refill time (5 seconds), a carbon dioxide gap of 7 mmHg, was hypotensive (MAP <60 mmHg), and anuric. POCUS (Mindray M8 Elite; Shenzhen Mindray Bio-Medical Electronics Co., Ltd., Shenzhen, China) was performed by the ICU team, which showed an ejection fraction of 30% (visual) and a low cardiac output with a left ventricular outflow tract velocity time integral (LVOT vti) of 10.4 cm. Mitral inflow Doppler revealed an E/A of 1.9 and an E/e’ of 6.0, with bilateral B lines noted on lung ultrasound. Her right ventricle was enlarged with paradoxical septal movement in diastole with a tricuspid annular plane systolic excursion (TAPSE) of 11.7 mm. The interatrial septum was also noted to be bowed to the right (Figure 1). Inferior vena cava (IVC) assessment revealed a maximum diameter of 2.40 cm, and right ventricular systolic pressure was estimated at 33 mmHg. Venous excess ultrasound examination revealed a portal vein that was >100% pulsatile (Figure 2). Given the POCUS findings and the pulsatile portal vein, fluid removal on CRRT was commenced (100 mL/hr), and an adrenaline infusion was started at 1 μg/kg/min.

By day 2 in the ICU, a total of 2400 mL of fluid was removed via CRRT. Fluid removal was well tolerated, with stable noradrenaline requirements and decreasing adrenaline dose. POCUS was repeated 24 hours after the first scan, which showed an approximately 100% pulsatile portal vein, with a 2.2 cm IVC (<50% collapsibility) and an E/A ratio of 2.5. Fluid loss was increased to 200 mL/hr, and the patient was subsequently extubated.

By the end of day 3 in the ICU, a total of 8.5 L of fluid was removed via CRRT. Despite her ongoing fluid removal, the noradrenaline infusion was gradually decreased and discontinued by the end of day 3. CRRT was discontinued after 72 hours in the ICU with significant clinical improvement, being weaned off both supplemental oxygen and vasoactive medication, and with normalization of her base excess and capillary refill time. She was eventually transitioned to the medical ward and started on intermittent hemodialysis.

## Discussion

POCUS monitoring in leptospirosis has unique advantages over standard monitoring parameters used in critically ill patients. Central venous pressure, while commonly monitored, has not been proven to be effective in determining fluid responsiveness and can be influenced by a wide variety of factors in ventilated patients [9,10]. Cardiac output monitors, although useful, may not be available in many limited-resource settings [11]. Useful hemodynamic information can also be provided by pulmonary arterial catheters (PACs), which can provide information on LAP and cardiac output. However, PACs are not in widespread use in many ICUs and may be associated with significant patient-related complications [12]. Advantages of POCUS include its availability, safety, repeatability, relatively low cost, and ability to provide data that would have previously required a combination of the monitors listed above. Additionally, unique features of POCUS monitoring include its ability to directly monitor end-organ congestion through portal vein pulsatility and to determine fluid tolerance [5,8]. 

In a South Indian study using myocardial strain imaging, biventricular systolic dysfunction was present in 30% of patients with leptospirosis [13]. Given the complex treatment implications, POCUS is well-suited to guide and monitor therapy in such cases. This pattern of biventricular dysfunction, characterized by reduced TAPSE and LVOT vti, was also observed in our case. In our patient, ECG and echocardiogram findings and positive troponins would have indicated myocardial involvement and a likely myocarditis.

To augment stroke volume, fluid therapy is often the first medication administered in the critically ill patient [14]. This should warrant prior consideration of LAP and fluid tolerance, which can be non-invasively determined using POCUS [5,15]. A high LAP would increase the risk of pulmonary edema and make fluid responsiveness unlikely. Markers of high LAP and fluid intolerance in our patient include a high E/A ratio, interatrial septal shift, paradoxical septal motion, distended IVC, and pulsatile portal vein [15]. Hence, further fluids were withheld, and inotropic and vasopressor therapy started instead. Of note, a multiplicity of parameters was used in this patient to determine her LAP. Given her young age, E/e’ ratio, a commonly used determinant of LAP, was normal, leaving us to rely on other parameters in combination (interatrial septal bowing, B lines, and E/A ratio; Figure 1) [15,16].

The other clinical challenge encountered in our patient was the presence of shock and a simultaneous fluid overload state. Fluid removal via CRRT has the potential to worsen shock and organ dysfunction by further compromising cardiac output. POCUS, specifically portal vein pulsatility, is a novel parameter that can provide a marker for pathological organ congestion and serve as a titratable guide for fluid removal [8,17,18]. Organ congestion is a pathological state that can lead to worsening acute kidney injury, reduced cardiac output, and mortality [19]. Although the clinical context is important, portal vein pulsatility can serve as an important guide to decongestion in many clinical scenarios [8,17,20-22]. Portal vein pulsatility is also easy to obtain, thus increasing its applicability.

Guided by portal vein pulsatility and POCUS, our patient had the safe removal of close to 9 L of fluids, which led to the resolution of her shock and pulmonary congestion, allowing her to be discharged from the ICU. The safety of fluid removal was confirmed with repeat POCUS after 24 hours, which showed persistent organ congestion (pulsatile portal vein) and an E/A ratio of >2, allowing for continued fluid removal.

The use of POCUS has the potential to provide significantly more hemodynamic data to clinicians at the bedside; however, there are limitations associated with its use. Novel parameters such as portal vein pulsatility have shown promise in early trials, primarily in patients after cardiac surgery and patients with cardiogenic shock, but further studies are required in patients with sepsis and septic shock [7,19]. Despite the ease of obtaining images, interpretation of portal vein and POCUS data can be challenging, and overreliance on a single parameter is not advised [15,23,24]. Accurate clinical decision-making would require a combination of multiple clinical and ultrasound data along with frequent reassessment, which can become labor-intensive and prohibitive.

## Conclusions

Given the complexities of leptospirosis treatment, POCUS can provide multiple hemodynamic parameters, which can prove useful in guiding care in leptospirosis patients. Further prospective studies are needed to assess the use of POCUS modalities in these patients. 
